# Comparison of the effect of NaOCL, curcumin, and EDTA on differentiation, proliferation, and adhesion of dental pulp stem cells

**DOI:** 10.1016/j.jobcr.2023.03.002

**Published:** 2023-03-10

**Authors:** Vahid Zand, Amin Salem Milani, Carolyn Primus, Marzie Aghazade, Hadi Mokhtari, Sabete Bagheri Sabzevar, Pardis Tehranchi

**Affiliations:** a Department of Endodontics,Faculty of Dentistry, Tabriz University of Medical Sciences, Tabriz, Iran; b The Dental College of Georgia at Augusta University, Primus Consulting, Consultant in Medical Device, Certified in New Product Development, USA; c Department of Oral Medicine Faculty of Dentistry, Tabriz University of Medical Sciences, Tabriz, Iran; d Department of Endodontics, School of Dentistry, Tabriz University of Medical Sciences, Tabriz, Iran; e Department of Operative and Esthetic Dentistry, Dental School, Tabriz Azad University of Medical Sciences, Tabriz, Iran

**Keywords:** NaOCl, Irrigants, Curcumin, 17% EDTA, Differentiation, Cell adhesion, Proliferation

## Abstract

**Introduction:**

This study examined the effect of 1.5% NaOCl, 17% EDTA, and curcumin on the proliferation, attachment, and differentiation of dental pulp stem cells (DPSCs) placed on the dentin specimens.

**Methods:**

MTT assay was performed to evaluate the proliferation of DPSCs on the dentin specimens treated with different concentrations of NaOCl, 17% EDTA, and curcumin (0.97–250 μM). Cell-adhering ability of DPSCs was tested via the LDH assay to calculate the attached DPSCs. In addition, the western blotting assay was performed to investigate the expression levels of fibronectin as a cell-adhesion marker and analyze the expressions level of differentiation markers, including DMP-1, OCN, ALP, and DSPP, to detect the odontogenic potential of hDPCs.

**Results:**

NaOCl had lower toxicity on DPSCs at lower concentrations (P < 0.001). The cytotoxicity of irrigants increased with increased dosage. The difference between the cell-adhesion ability of NaOCl and curcumin was not significant (∼4.4 MU/mL), whereas EDTA (∼3.8 MU/mL) exhibited the lowest release of LDH and less damage to hDPSCs. Regarding fibronectin expression, the pattern differed between irrigants in inducing cell adhesion. NaOCl increased fibronectin expression more than EDTA and curcumin. All the treated groups upregulated the expression of DSPP, DMP-1, OCN, and ALP compared to the control group, in which NaOCl showed a higher effect on the overexpression of differentiation markers.

**Conclusion:**

The results showed that all the tested irrigants could be used in regenerative endodontic treatment. However, as an herbal-based and biocompatible irrigant, curcumin exhibited fewer adverse effects than NaOCl and EDTA.

## 1 Introduction

The process of regeneration of human cells, tissues, and organs, called regenerative medicines, is a novel therapeutic application aiming to replace damaged, diseased, and missing structures.^1^^,^^2^ Dental pulp stem cells are similar to bone marrow stem cells and possess an outstanding ability to produce dentin-like structures and differentiate into odontoblastic and osteoblastic phenotypes.^3^^,^^4^

Irrigating solutions are used in regenerative endodontic therapy with cytotoxic effects on DPSC.^5^ Sodium hypochlorite (NaOCl) is one of the most common and effective endodontic irrigants used as an antimicrobial medicament. The antibacterial potential of NaOCl can be associated with reducing the surface tension of the solution by being a fat solvent, breaking bonds between carbon atoms, degrading dentin collagen, and finally influencing the primary structure of proteins.^6^^,^^7^

Nevertheless, toxic effects on periapical tissue and subsequent delay in wound healing, extensive tissue damage, and unpleasant smell and taste are crucial drawbacks of using NaOCl in clinical cases and basic research studies of regenerative therapy.^8^^,^^9^ EDTA is another irrigant that induces growth factors released from the dentin matrix, causing differentiation of the stem cells into odontoblasts in the dentin structure.^10^ Better survival of stem cells is another advantage of using EDTA in regenerative endodontic therapy attributed to its potential in demineralizing superficial dentin, increasing adhesion of DPSCs, and releasing dentin endogenous growth factors.^11^^,^^12^

Using herbal medicines as proliferative and differentiative agents can be non-toxic and cost‐effective in stem cell-based therapies.^13^ Curcumin [diferuloylmethane or (1E,6E)-1,7-bis(4-hydroxy-3-methoxyphenyl)-1,6heptadiene-3,5-dione] as an herbal-based plant is a natural and active polyphenolic hydrophobic product isolated from the rhizomes of *Curcuma longa*.^13^^,^^14^ Differentiation of stem cells is one of the critical advantages of curcumin on osteolysis, osteoporosis, and osteosarcoma.^1^ It has been shown that curcumin increases osseous mineral density and bone remodeling.^13^^,^^15^

This study investigated the effect of three irritants, including curcumin as a plant-based component, NaOCl, and 17% EDTA on the proliferation, attachment, and differentiation of DPSCs plated on dentin specimens.

## 2 Materials and methods

### 2.1 Materials

MTT [3-(4, 5-dimethylthiazol-2-yl)-2, 5-diphenyltetrazolium bromide] powder was provided by Sigma Chemical Co. (St. Louis, MO, USA). Lactate dehydrogenase (LDH) activity assay kit (Catalog Number MAK066) was purchased from Sigma Aldrich (Sigma Aldrich, USA). Primary and secondary antibodies were supplied by Santa Cruz Biotechnology and Abcam companies (USA, England). NaOCl and curcumin were provided by Nik Darman Asia and Safir Azma companies (Iran), respectively. All cell culture materials were supplied by appropriate, commercially available suppliers.

### 2.2 Dentin specimen preparation

This study was approved by the ethics committee of the Tabriz University of Medical Sciences, Dental Faculty, Tabriz, Iran. Third molar teeth diagnosed with vital pulp but with an indication for extraction were collected from the clinics of the Tabriz University of Medical Sciences upon informed consent. Dentin specimens, cut in 1-mm^3^ cubes, from human molars, extracted using a low-speed diamond saw (Isomet, Buehler, Lake Bluff, IL, USA) under constant irrigation with phosphate buffered saline (PBS). The specimens were sterilized by ethylene oxide gas. EDTA 17% solution (Nik Darman, Tehran, Iran) was used to remove the smear layer in specimens. All the specimen discs were washed with PBS five times and placed in one layer in 60-mm Nunclon Sphera culture dishes. The specimen samples were randomly divided into four groups to be treated based on 17% EDTA(ED), curcumin(Cu), NaOCl(Na). The fourth group was considered a control with no irrigant treatment.

### 2.3 Cell culture

hDPSCs provided by the Iranian Biological Resource Center (IBRC, Iran) were suspended in RPMI (Roswell Park Memorial Institute, Gibco, USA) culture medium supplemented with heat-inactivated 10% fetal bovine serum (FBS, Gibco, USA) and 100-U/mL penicillin, 100-μg/mL streptomycin (Gibco, USA), and incubated at 37 °C in a humidified atmosphere of 5% CO_2_ and grown to 80% confluency.

### 2.4 Cell viability assay

The effect of NaOCl, EDTA, and curcumin on cell viability was assessed using an MTT assay. DPSCs were seeded at 10 × 10^3^ cells/well on the dentin slices incubated with NaOCl, EDTA, and curcumin into a sterile 96-well plate. Differentiation medium incubated with DPSCs and dentin specimen was considered a positive control and the untreated cells as a negative control group. The cells were incubated at 37 °C in 5% CO_2_ for seven days. On day 7, 100 μL of MTT stock solution (5 mg/mL) was added to DPSCs to achieve a final concentration of 0.5 mg/mL and incubated for 3 h at 374 °C. At the end of the incubation period, the medium was removed, and DMSO was added to dissolve formazan crystals. A spectrophotometric Microplate Reader (Thermo Fisher Scientific, USA) was used to read the optical density at 570 nm by a spectrophotometric Microplate Reader.

### 2.5 Immunoblotting assay

Dentin slices were placed in 6-well plates in a single layer after five times of washout with PBS. Then, six groups, including intact cells as a negative control, cell + dentin, cell + dentin + NaOCl, cell + dentin +17% EDTA, cell + dentin + curcumin, and cell + dentin + differentiation medium were used to investigate the effect of dentin specimen on the dental stem cells. Harvested hDPCs were washed with cold PBS and then lysed using ice-cold lysis buffer (2 mM EDTA, 1% Triton X-100, 1 mM sodium orthovanadate, 25 mM HEPES, 0.1 mM NaCl, and 25 mM NaF). The collated protein lysate was centrifuged at 12000 g for 20 min at 4 °C and measured via a Bradford protein assay kit. The defined protein lysate was examined using 10% SDS-PAGE and transferred to the polyvinylidene fluoride or polyvinylidene difluoride (PVDF) membrane. After blocking the membrane at room temperature using 5% non-fat milk for 1 h, primary antibodies were applied to incubate the proteins overnight at 4 °C. The primary antibodies applied were OCN (sc-30044), DMP-1 (sc-73633), DSPP (sc-73632), β-Actin (sc-47778), fibronectin (ab2413), and ALP (sc-271431). Next, secondary antibodies conjugated with peroxidase were incubated with a washed membrane. Protein expression was pictured with a Fuji radiographic film (Fuji Photo Film Co., Tokyo). Antibody against β-actin was used as a loading control (MAC066, Sigma, USA).

### 2.6 Lactate dehydrogenase (LDH) assay

Dentin specimens were rinsed with NaOCl, EDTA, and curcumin, followed by seeding hDPSCs on the dentin specimens in 6-well plates and incubated for 7 days at 37 °C and 5% CO_2_. An MAK066 lactate dehydrogenase permeability assay kit (Sigma Aldrich, USA) was used to measure the cytotoxicity of the irrigants. Thirty minutes of incubation, the fluorescence intensity was estimated at 490 nm with a multi-well plate reader to determine the amount of attached DPSC. The percentages of unattached DPSC were calculated based on: Unattached DPSCs (%) = (experimental absorbance value – low control absorbance value)/(high control absorbance value – low control absorbance value)*100. The percentage of unattached cells was subtracted from the total cells and designated as the percentage of attached cells.

### 2.7 Statistical analysis

All the experiments were repeated in triplicate under three independent conditions. The data were presented as the standard error of the mean (SEM), and differences were significant at P < 0.05. All analyses were carried out by GraphPad (Version 6, California, USA) and SPSS 21.0 (SPSS Inc., Chicago, IL, USA) software. To compare the level of differentiation and proliferation of stem cells between the studied groups, a one-way analysis of variance or non-parametric Kruskal-Wallis test was used. The chi-square test also was used to compare the level of adhesion between the studied groups.

## 3 Results

### 3.1 Cell proliferation

**Fig. 1:**
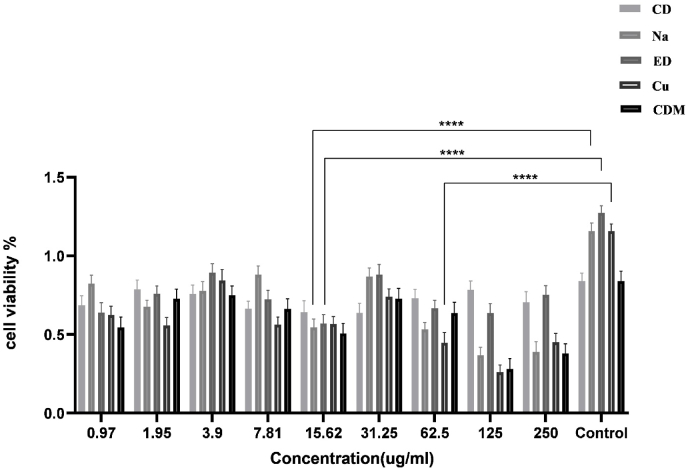
The effect of different concentrations of NaOCL, EDTA17%, and Curcumin on the proliferation and variability of DPSCs.

MTT assay was used to compare the proliferation of DPSCs in the presence of dentin specimens after treatment with NaOCl, EDTA, and curcumin at various concentrations ranging from 0.97 to 250 μM on day 7. Accordingly, the means and standard deviations of curcumin, NaOCl, and EDTA are presented in Table 1, and its comparative chart is shown in Fig. 1. We showed that NaOCl had lower toxicity on DPSCs at lower concentrations (P < 0.001). In contrast, cell growth decreased considerably at higher concentrations, reaching 40% compared to the control group (100%). Regarding EDTA toxicity on the proliferation of DPSCs, the cytotoxic effect of EDTA on the survival of DPSCs was lower than NaOCl (P˂0.002). Curcumin displayed a similar pattern as NaOCl in reducing the viability of DPSCs at the evaluated concentrations. Curcumin was more effective at lower concentrations on the proliferation of DPSCs (P < 0.001); however, at higher concentrations, it decreased cell viability more than other irrigants. The cell + dentin + differential media was considered a positive control.

**Table 1:** Mean and standard deviation of different concentrations of NaOCL (Na), EDTA 17% (ED), curcumin (Cu), control group with dentin and control group with dentin and differentiation medium.

investigated groups	Concentrations	Mean	Standard deviation	P-value
CD	.97	.68575	.059028	.005
	1.95	.78725	.059315	
	3.90	.75775	.055955	
	7.81	.66525	.047458	
	15.62	.64275	.071658	
	31.25	.63800	.062032	
	62.50	.73050	.057041	
	125.00	.78325	.057262	
	250.00	.70450	.068651	
Na	.97	.82325	.055157	<.001
	1.95	.67775	.041015	
	3.90	.77825	.057783	
	7.81	.88100	.054772	
	15.62	.54475	.053848	
	31.25	.87025	.052867	
	62.50	.53275	.043950	
	125.00	.36925	.049149	
	250.00	.38950	.064645	
ED	.97	.63950	.063883	**<.001**
	1.95	.75975	.049507	
	3.90	.89275	.058506	
	7.81	.72375	.057679	
	15.62	.56925	.056929	
	31.25	.88150	.064091	
	62.50	.66850	.047983	
	125.00	.63525	.061727	
	250.00	.75175	.059152	
Cu	.97	.62375	.056192	**<.001**
	1.95	.55900	.050146	
	3.90	.84475	.067460	
	7.81	.56225	.048541	
	15.62	.56600	.049645	
	31.25	.74100	.049078	
	62.50	.44900	.063755	
	125.00	.26125	.045850	
	250.00	.45100	.057428	
CDM	.97	.54600	.065366	**<.001**
	1.95	.72675	.061841	
	3.90	.74875	.060901	
	7.81	.66275	.065040	
	15.62	.50700	.062134	
	31.25	.72775	.066385	
	62.50	.63650	.069936	
	125.00	.28000	.067424	
	250.00	.38050	.062035

### 3.2 Cell attachment analysis by measuring LDH and fibronectin

**Fig. 2:**
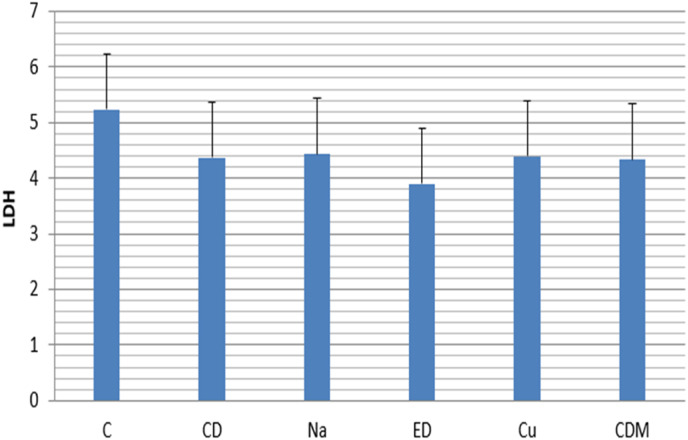
DPSCs attachment to the dentin specimen treated. For each group, three independent experiments were performed (n = 3).

The amount of LDH release was determined to assess the percentage of unattached and attached DPSCs to dentin specimens. The means and standard deviations of LDH and Fibronectin are presented in Table 2. Accordingly, the comparative chart is shown in Fig. 2 and Fig. 3a. The results showed that dentin treated with EDTA (3.8 MU/mL) increased the attachment of DPSCs significantly compared with the control group (Fig. 2). Moreover, NaOCl (4.4 MU/mL) and curcumin (4.4 MU/mL) had a similar effect on the attachment of DPSCs. It can be seen that all the treatment groups exhibited a lower amount of LDH release (P < 0.435) compared to intact cells as a negative control.

**Table 2:** Mean and standard deviation of LDH and fibronectin in different groups Na, ED, Cu, CD and CDM.

Investigated groups	LDH		Fibronectin	
	Mean	Standard deviation	*Mean*	Standard deviation
C	5.2333	.75719	2418.14	251.53
CD	4.3667	.66583	2082.47	1053.83
Na	4.4333	.70238	9321.83	942.23
Ed	3.9000	.30000	5240.28	397.15
Cu	4.4000	1.04403	7068.18	2064.29
CDM	4.3333	.73711	9256.56	1922.08
P-value	.435		<.001

**Fig. 3a:**
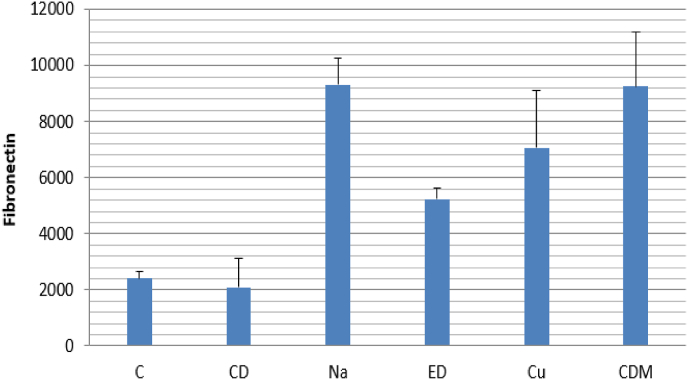
fibronectin expression level as a cell adhesion marker on DPSCs.

The protein expression level of fibronectin was measured via immunoblotting assay to investigate the attachment of HDPSCs on dentin specimens (Fig. 3b). Based on the results in Fig. 3a, fibronectin level was upregulated in all the treatment groups compared to the control group. NaOCl increased fibronectin expression level more than threefold, which was higher than other irrigants (P < 0.001). Furthermore, fibronectin expression increased after treatment with curcumin (P < 0.014). EDTA (P < 0.273) exhibited the lowest effect on fibronectin overexpression compared to NaOCl and curcumin.

**Fig. 3b:**
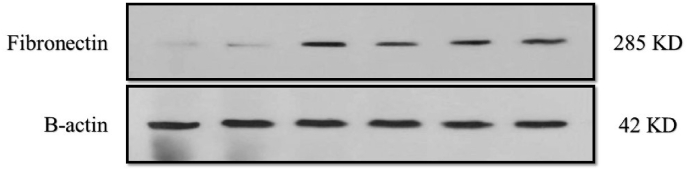
immunoblotting assay to evaluate the attachment of HDPSCs on dentin specimens.

### 3.3 Cell differentiation analysis using western blotting

The means and standard deviations of differentiation markers are presented in Table 3. Accordingly, the comparative chart is shown in Fig. 4a. To investigate the effects of different irrigants on protein expression of differentiation markers, DPSCs were incubated with NaOCl, EDTA, and curcumin (Fig. 4b). Untreated cells were negative control, and cells treated with the differential medium were positive control. The expression of dentin sialophosphoprotein (DSPP), alkaline phosphatase (ALP), osteocalcin (OCN), and matrix protein-1 (DMP-1) increased after treatment with NaOCl, EDTA, and curcumin, among which NaOCl showed a significant difference in protein expression compared to other irritants (Fig. 4a). However, curcumin showed the lowest protein expression in differentiation markers compared to NaOCl and EDTA.

**Table 3:** Mean and standard deviation of differentiation markers DSPP, ALP, OCN and DMP1 in different groups Na, ED, Cu, CD and CDM.

Investigated groups	DSSP		ALP		OCN		DMP_1		B_actin	
	Mean	standard deviation	Mean	standard deviation	Mean	standard deviation	Mean	standard deviation	Mean	standard deviation
C	2583.94	290.74	3730.49	1716.36	4159.82	1961.60	2476.04	386.78	12444.79	1131.74
CD	2979.89	1390.57	3328.54	723.69	3541.76	1025.63	2227.32	900.85	12725.91	655.33
Na	9526.78	2812.83	10922.84	3314.44	12657.48	7446.04	10476.33	1385.04	12698.65	760.66
Ed	8151.30	1218.92	7326.08	3228.82	10963.49	6450.14	6628.41	832.75	12543.00	745.30
Cu	6085.07	1652.39	5976.28	2848.87	8138.84	4205.68	3282.75	2176.33	12124.82	1067.87
CDM	11595.08	2639.78	10825.87	1761.29	14900.65	6288.52	11091.24	1112.99	12418.18	916.89
P-value	<.001		.007		.100		<.001		.964

**Fig. 4a:**
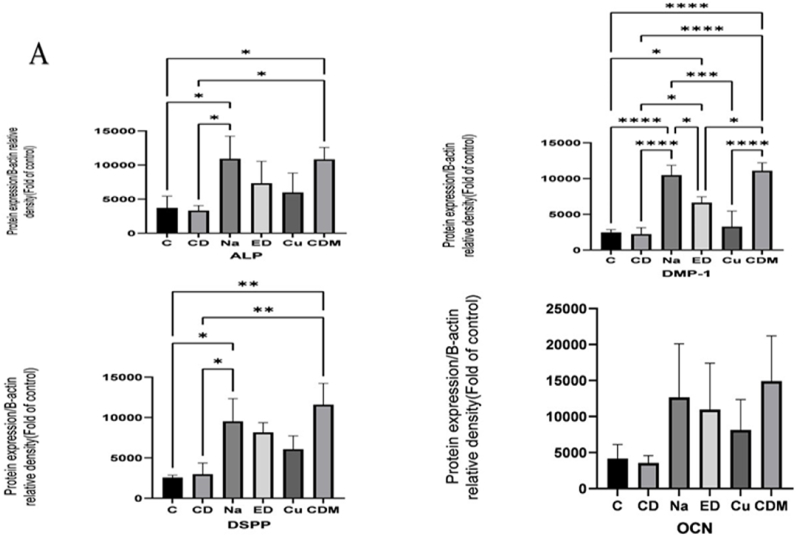
Western blotting analysis of differentiation markers.

**Fig. 4b:**
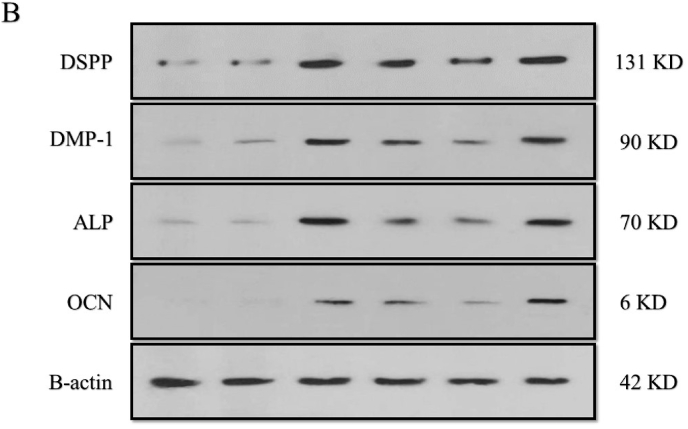
The protein expression of differentiation markers.

## 4 Discussion

As an alternative treatment, pulp revascularization results from dentinal wall thickness and induces root canal development of immature permanent teeth with necrotic pulps.^10^^,^^16^ Three common protocols are carried out in the root canal space, including 1) root canal disinfection, 2) induction of bleeding into the canal as a scaffold for tissue ingrowth, 3) using mineral trioxide aggregate to cover the root canal.^10^^,^^17^

Sterilization and eliminating bacteria from the root canal entails using appropriate irrigants with antimicrobial properties.^5^^,^^18^ Optimizing the biological environment of the root canal via irrigations is another step in regeneration therapy.^19^ The next step in endodontic regeneration therapy is optimizing cells attached to the dentin surface after migration into the root canal to differentiate and proliferate into odontoblast-like cells.^10^^,^^20^

This study investigated the effect of curcumin, EDTA, and NaOCl on the attachment, differentiation, and proliferation of DPSCs on dentin specimens. We used MTT assay to determine the proliferation effect at different concentrations on DPSCs. NaOCl, curcumin, and EDTA had cytotoxic effects on the DPSCs at various concentrations and exhibited increased cytotoxicity gradually with an increase in concentration. The results showed that NaOCl, 17% EDTA, and curcumin had a dose-dependent effect on the survival of DSPCs.

Consistent with the present study, Liu et al. investigated the cytotoxic effect of NaOCl on the mesenchymal stem cells at different concentrations. They suggested that NaOCl decreased the cell viability of dental stem cells in a time- and concentration-dependent manner. They concluded that NaOCl should be used with caution in endodontic therapy as it reduces dental stem cell proliferation, differentiation, and survival.^7^ In another study, Samiei et al. investigated the cytotoxic effect of curcumin alone and in combination with calcitriol on DPSCs by the MTT assay. They showed that the vitality of DPSCs decreased after treatment with high concentrations of curcumin^15^. Stimulating DPSCs attachment to the root canal is crucial in regenerative endodontic therapy. Cell attachment is mediated by the correct geometry of adsorbed proteins deposited either by the cells themselves or adsorbed from body fluids naturally.^10^^,^^21^ Fibronectin as a preabsorbed protein has an essential role in cell adhesion, and its increased adhesion enhances cell attachment.^10^^,^^22^ In this study, we used the western blotting assay to calculate the protein expression level of fibronectin after treating DPSCs with NaOCl, curcumin, and EDTA to show the amount of cell attachment on dentin specimens. All the mentioned irrigants showed an increase in fibronectin expression compared to the control group, of which NaOCl was more efficient in inducing cell attachment.

LDH activity is another factor used to evaluate cell attachment on dentin specimens after treatment with the irrigants mentioned above. Quantification of the amount of LDH released can be used as a marker to assess damaged tissues. Following tissue damage, cells release LDH into the bloodstream, resulting in the loss of the cytoplasm and consequently cell death.^23^ The amount of LDH was measured by colorimetry assay, and the results showed that these irrigants decreased the LDH release from DPSCs. EDTA was more effective in reducing cell death compared to the control group.

Differentiation is the next step to show the effect of various irrigants on the regeneration of odontoblasts after the attachment of DPSCs on the dentin specimens.^24^ In the present study, odontogenic differentiation was examined by evaluating the protein expression level of OCN, DSPP, DMP-1, and ALP from hDPSCs. DSPP is a gene family of small, integrin-binding ligand, an N-linked glycoprotein cleaved into dentin sialoprotein (DSP) and dentin phosphoprotein (DPP). DSP and DPP have an active role in dentinogenesis. Increasing the expression level of DSPP resulted in the overexpression of other odontogenic genes and mineralization in stem cells.^12^ We showed that the protein expression levels of the DSPP and DMP-1 upregulated at the early stage of odontogenesis. As a marker for odontoblast and osteoblast differentiation, DSPP caused a significant growth in expression after treatment with NaOCl, 17% EDTA, and curcumin, respectively. DMP-1 activates DSPP gene transcription by regulatory functions. DMP-1 binds to the DSPP promoter in early odontoblast differentiation, explaining the harmonized expression of DSPP and DMP-1 in osteocalcin^13^.

In this study, the expression of DMP-1 increased upon treatment with NaOCl, EDTA, and curcumin, suggesting that these irrigants promoted the differentiation of the attached DPSCs on the dentin specimens to odontoblast/osteoblast cells. However, NaOCl enhanced the expression of DMP-1 more than other irrigants compared to the negative control group. Cell + dentin + differentiation media as a positive control was effective in the expression of both DSPP and DMP-1. ALP as an early osteogenic/odontogenic marker increases osteogenic and odontogenic differentiation of DPSC.^15^ Our findings showed an increase in the expression of ALP in all the treated groups, of which NaOCl enhanced the expression of ALP more than curcumin and EDTA compared to the negative control group. OCN can be considered a marker to confirm odontogenic differentiation and osteogenic capability. It has been demonstrated that the overexpression of DMP-1 induces the expression of osteogenic genes such as OCN.^3^

Additionally, in osteogenic conditions, antisense DMP-1 blocks OCN synthesis, indicating that OCN is a downstream gene, regulating its expression by DMP-1. The present findings are consistent with these observations, demonstrating that the protein level of OCN upregulated similarly to DMP-1 expression in DPSCs. Consistent with the present study, Son et al. assessed the curcumin effect on the differentiation of mesenchymal stem cells (C3H10T1/2). Their findings showed that curcumin enhanced the expression of marker genes of osteoblastic differentiation by inducing ATF6 expression. Moreover, curcumin developed osteoblast differentiation by increasing the expression of osteogenic genes, including ALP.^13^

## 5 Conclusion

This study showed that DPSCs treated with NaOCl, 17% EDTA, and curcumin had significant higher proliferation in a dose-dependent manner. Adhesion of DPSCs on dentin specimens was induced by irrigants. These irrigants can be recommended in endodontic regeneration treatment, of which curcumin, as a natural material.

## 6 Limitations

This study only considers the effect of curcumin, EDTA, and sodium hypochlorite at the cellular level. Therefore, further analyses are needed to investigate curcumin side effects on the proliferation, adhesion, and odontoblastic/osteoblastic differentiation of DPSCs in regenerative endodontic practice compared to NaOCl and 17% EDTA solution. Furthermore, future studies can be done to evaluate the scaffolds, bone grafts, and membranes containing curcumin, EDTA, and sodium hypochlorite and their combination in the procedures of immature teeth regeneration and guided regeneration of tissue and bone.
